# Development and validation of a short form for the Raven's Coloured Progressive Matrices using a machine learning approach

**DOI:** 10.1111/bjdp.12542

**Published:** 2025-01-23

**Authors:** Charles Chiu Hung Yip, Terry Tin‐Yau Wong, Brandon Hoi Dick Wong, Lucy Shih‐Ju Hsu

**Affiliations:** ^1^ Department of Psychology The University of Hong Kong Pokfulam Hong Kong

**Keywords:** developmental research, fluid intelligence, general cognitive ability, Raven's Coloured Progressive Matrices, short form, young children

## Abstract

Raven's Coloured Progressive Matrices (CPM) is a widely used assessment tool for measuring general cognitive ability in developmental and educational research, particularly in studies involving young children. However, administering the full set of the 36‐item CPM can be burdensome for young participants, hindering its practicality in large‐scale studies and reducing research efficiency. In the current study, a short form of the CPM was developed based on a sample of preschoolers (*n* = 336, mean age = 5.8 years) using penalised regression, a machine learning approach that allows for variable selection. The resulting 12‐item CPM short form demonstrated a very strong correlation with the total score of the 36‐item full form (*r* = .94). Further investigations into the short form's item stability, content validity, and concurrent validity collectively supported its psychometric properties as a reliable and valid alternative to the full form. The significance of the CPM short form is also discussed.

## Graphical abstract

[Correction added on 26 June 2025: Graphical Abstract has been included in this version.]

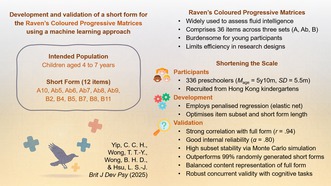

**Statement of contribution**

**What is already known on this subject?**

- Raven's Coloured Progressive Matrices (CPM) is a widely used assessment tool in developmental and educational research of young children.
- Administration of the full 36‐item CPM is time‐consuming and burdensome for young participants.
- Despite the longstanding and extensive use of CPM, no short form has been developed for children.

**What the current study adds?**

- A 12‐item CPM short form was developed and validated with children aged 4–7 years.
- Multiple validation methods supported the CPM short form as a valid and reliable alternative to the full form.

## BACKGROUND

Raven's Coloured Progressive Matrices (CPM) is a widely used assessment tool in developmental and educational research, particularly in studies involving young children. It is part of the Raven's Progressive Matrices series, which also includes the Standard Progressive Matrices (SPM) for the general population and the Advanced Progressive Matrices (APM) for individuals with higher cognitive abilities (Raven et al., 1998). Collectively, this series of tests was designed to evaluate nonverbal and analogical reasoning skills, also referred to as tests of “observation and clear thinking” (Raven et al., 1998, p. G57). In research with young children, the CPM is commonly used as a proxy measure for constructs such as fluid intelligence (Memisevic et al., 2023; Ouyang et al., 2023), nonverbal reasoning (Alhamdan et al., 2023; Ng et al., 2021), and general cognitive ability (Carvalho et al., 2022; Gofer‐Levi et al., 2014). The CPM consists of 36 items, which are divided into three 12‐item sets (A, Ab, and B). Since the CPM is language‐independent, it can be used to test a wide range of populations, including young children, individuals with intellectual impairments, and the elderly. It is also adaptable to different cultural contexts. Past research has garnered substantial support for its strong psychometric properties, as evidenced by its significant correlations with theoretically related constructs (e.g., *r* = .40 with mathematics ability; Agnoli et al., 2012; *r* = .41 with overall achievement; Kazem et al., 2007), a high degree of test–retest reliability (e.g., *r* = .84; Costenbader & Ngari, 2001), and good internal consistency (e.g., Cronbach's *α* ≥ .76, split‐half reliability ≥ .89; Cotton et al., 2005) in population‐based studies across different cultures. These qualities contribute to its enduring popularity among researchers.

Although the 36‐item CPM offers several advantages as a well‐studied psychometric instrument, a limitation lies in the time needed to administer the full set of items. This issue was also highlighted in previous studies examining the 60‐item SPM and the 48‐item APM (Arthur & Day, 1994; Bilker et al., 2012; Bors & Stokes, 1998; Langener et al., 2022). This limitation is particularly pertinent to the CPM for testing young children due to their limited attention span, where prolonged testing may induce inattention and reduce motivation, which could compromise the validity of the measures in the assessment batteries (Blazek & Siegel, 2024; Borgers et al., 2000). This is also in line with previous reports of fatigue and exhaustion related to administering the full 36‐item CPM (Van de Vijver & Brouwers, 2009). More importantly, as the full 36‐item CPM requires 15–30 minutes to administer (Ammawat et al., 2022; Costenbader & Ngari, 2001), its lengthy design may restrict researchers from including additional measurements of interest in their studies, leading to inefficiencies in research designs. The resource‐intensive nature of administering the full CPM, such as the recommended individual or small group administration procedures (Raven et al., 1998), further hinders its practicality for large‐scale studies, which are valuable in developmental and educational contexts. Given these limitations and implications, there is a pressing need to develop a short form for the CPM that maintains its psychometric properties while providing a more efficient alternative to the full version.

### Existing short forms of Raven's Progressive Matrices

Previous studies have developed shorter versions of the APM and SPM for adult and school‐age populations. For adult populations, Arthur and Day (1994) created a 12‐item APM based on item difficulties and item‐total correlations, while Bors and Stokes (1998) developed another 12‐item APM using item‐total correlations and item factor scores. Additionally, Bilker et al. (2012) used an iterative algorithm to create two 9‐item SPM short forms. For school‐aged populations, Langener et al. (2022) used penalised regression models and item response theory (IRT) to develop two 15‐item SPM short forms for two age groups (i.e., 9–12 and 13–16 years), respectively. The short forms developed with penalised regression showed good psychometric properties and outperformed those created with IRT. Despite these efforts in developing abbreviated forms of APM and SPM, they are not suitable for studies involving young children, as their ability to form comparisons and reason by analogy has not matured, which may lead to unreliable and psychologically invalid results in younger samples (Raven et al., 1998).

There are currently two abbreviated versions of the CPM. In a norming study, Smits et al. (1997) generated CPM norms with an older adult sample using a 24‐item CPM that included Sets A and B but excluded Set Ab, as previous research suggested Set Ab had limited discriminative power among older adults (Levinson, 1959). However, these findings based on older adult samples cannot be directly applied to early childhood populations due to potential differences in cognitive development and response patterns (Raven et al., 1998). Lúcio et al. (2019) made another attempt to reduce the CPM length by selecting the first six items from each set. They developed an 18‐item reduced form based on a confirmatory factor model using a sample with a mean age of 4.75 years (*SD* = 0.58 years). However, it is important to note that since items within CPM sets are arranged in ascending order of difficulty, items from the first half of each set may be considered too easy for older children. In fact, Smirni (2020) conducted a study categorising CPM into three factors and found that children as young as six years old could solve all items in one of the factors, namely simple pattern completion, which accounted for 11 out of the 18 items in Lúcio et al.'s (2019) reduced form. This suggests that Lúcio et al.'s (2019) reduced form may have limited discriminative power and generalisability for assessing older children.

### The current study

Taken together, prior studies have documented various efforts to shorten Raven's Progressive Matrices, including the CPM, to assess general cognitive ability more efficiently. However, to our best knowledge, none of the existing short forms of the CPM represented all the items from the full form that are suitable for children across a wider age range (i.e., aged 4–7 years). This is surprising given the longstanding and extensive use of the CPM to assess child participants across different fields of study, most notably in developmental and educational psychology. Hence, the goal of this study was to develop a short form of the CPM that maintains the psychometric properties of the full version and effectively predicts general cognitive abilities in preschoolers and early elementary school students.

## METHODS

### Participants

A total of 336 preschoolers (163 males) were recruited from seven local kindergartens in Hong Kong as part of a longitudinal study. The participants had a mean age of 5 years 10 months (*SD* = 5.5 months). All participants were native Cantonese speakers and attended Cantonese‐medium schools.

### Procedures

The current study was part of a longitudinal research project on language and cognitive development that was approved by the Human Research Ethics Committee of the authors' university (reference number: EA210486). Invitation letters were sent to kindergartens in Hong Kong, and consent forms were subsequently distributed to the parents. Upon parental consent, children were invited to attend individual assessment sessions at their kindergartens. All assessments were administered individually by trained research assistants and undergraduate students.

### Measures

#### Raven's Coloured Progressive Matrices

Raven's Coloured Progressive Matrices (CPM) was administered to assess children's non‐verbal reasoning ability, which refers to the ability to solve non‐verbal problems based on visual processing and inductive inferences (Raven et al., 1998). The CPM has 36 items in total, which were presented continuously as three 12‐item sets (Sets A, Ab, B). In each item, children were presented with a patterned diagram with a missing part and were asked to pick one of the six response choices that they thought would complete the pattern. Two practice items (i.e., A1 and A2) were provided with verbal feedback to ensure task comprehension and were excluded from subsequent analyses. Each correct response was awarded one point, with higher scores indicating better non‐verbal reasoning ability. The highest possible score on this task was 34. This measure showed good internal consistency in the current sample (Cronbach's *α* = .80).

#### Concurrent validity indicators

##### Verbal working memory span

Verbal working memory span was assessed by the backwards digit span (BDS) task. In each trial, the experimenter presented a sequence of single‐digit numbers (i.e., digits) at a pace of one number per second. The child participant was then asked to immediately repeat the sequence in reverse order. The task had seven levels, with each level having two items of the same number of digits. The number of digits started from two at the first level and increased by one digit at each subsequent level. The task was terminated when children failed to accurately reproduce both items at a level. Before the task began, one practice trial was provided with verbal feedback to ensure task comprehension. Each correct response was awarded one point, with higher scores indicating greater verbal working memory span. The highest possible score on this task was 14. This measure showed good internal consistency in the current validation sample (Cronbach's *α* = .76).

##### Inhibitory control

Inhibitory control was assessed by the Head Toes Knees Shoulders (HTKS) task (McClelland et al., 2007; Ponitz et al., 2009). The task comprised two parts, both requiring children to give opposite behavioural responses to the experimenter's instructions. In the first part, children needed to touch their heads when they were asked to touch their toes, and vice versa. In the second part, adding onto the rules from the first part, children needed to touch their knees when they were asked to touch their shoulders, and vice versa. Each part consisted of 6 practice trials and 10 test trials. Children were awarded two points for each correct response on their first attempt, one point for each correct response following an incorrect response (i.e., self‐correction), and no points for incorrect or no responses. Higher scores indicated better inhibitory control. The highest possible score on this task was 40. This measure showed good internal consistency in the current validation sample (Cronbach's *α* = .93).

##### Receptive vocabulary knowledge

Receptive vocabulary knowledge was assessed by the 65‐item Hong Kong Cantonese Receptive Vocabulary Test (CRVT; Lee et al., 1996). In each item, children listened to a target word (e.g., car, study, half) and were asked to point to the corresponding picture out of four options. The options included the correct choice, a phonological distractor, a semantic distractor, and an unrelated distractor. Before the task began, two practice trials were provided with verbal feedback to ensure task comprehension. Children were awarded one point for each correct response, with higher scores indicating better receptive vocabulary knowledge. The highest possible score on this task was 65. This measure showed good internal consistency in the current validation sample (Cronbach's *α* = .70).

##### Rapid automatised naming

Rapid automatised naming (RAN) was assessed with a RAN task of objects. The task consisted of 25 objects arranged in five rows, with each row containing the same five objects (i.e., bread, plane, frog, bike, sun) in a random order. Children were instructed to name all the objects from left to right and top to bottom as quickly as possible. The time taken to name all objects and the number of errors were recorded. Task performance was measured using the inverse efficiency score (IES), calculated by dividing the time taken by the proportion of correct responses. Lower scores indicated higher RAN efficiency.

###### Penalised regression

Penalised regression, also known as regularised regression, is a method widely used in machine learning to reduce overfitting of regression models, thereby improving model predictiveness and interpretability. This is achieved by adding penalty terms to the residual sum of squares to be minimised in the regression models. Different penalised regression methods, which differ in their penalty terms, exist to achieve different effects. Two methods are useful for the current purpose of short form development, including lasso regression, which adds a penalty term to minimise the absolute values of the model coefficients (Tibshirani, 1996), and ridge regression, which adds a penalty term to minimise the squared values of the coefficients (Hoerl & Kennard, 1988).

Both lasso and ridge regression have their advantages and disadvantages. While both methods involve shrinking model coefficients towards zero through penalty terms, lasso regression uniquely allows for variable selection by forcing some coefficients to become exactly zero (Tibshirani, 1996; Yang & Wen, 2018). However, lasso regression can encounter challenges with multicollinearity (Bondell & Reich, 2008; Zhu et al., 2015), which may be a concern in the case of CPM due to the expected similarity between certain items (Raven et al., 1998; Smirni, 2020). In contrast, based on the analyses of multiple experimental datasets, ridge regression can address multicollinearity more effectively than lasso regression (Hoerl & Kennard, 1988; Yang & Wen, 2018). To combine the benefits of both variable selection and multicollinearity handling, Zou and Hastie (2005) introduced elastic net regression. This approach integrates both lasso and ridge penalty terms into the regression model, striking a balance between selecting relevant variables and appropriately shrinking coefficients in the presence of correlated predictors.

In elastic net regression, the relative influence of lasso and ridge regressions can be adjusted by a mixing parameter (*α*), which ranges from 0 to 1, and is applied to the respective penalty terms. When *α* is set to 0, only the ridge penalty term is added, whereas when *α* is set to 1, only the lasso penalty term is added. Besides, a penalty parameter (*λ*) is assigned as a weight to the penalty terms to control their strength and, thus, determine the degree of shrinkage applied to the predictors in the model. In general, higher *λ* values push more coefficients towards zero and result in a simpler model with fewer predictors, while smaller *λ* values allow more coefficients to remain non‐zero and lead to a more complex model.

####### Short form development with penalised regression

The current study employed penalised regression to develop a short form for the CPM, where a linear model of the total score regressed on individual test items was fitted with the elastic net to reduce the number of model predictors. Through this approach, items that account for little unique variance in the total score with small coefficients in the regression model would be eliminated. This design builds upon the successful application of the same approach by Langener et al. (2022) in developing two SPM short forms for children and adolescents. These SPM short forms exhibited strong correlation with the full form (*r*s > .89), acceptable to good internal consistency (Cronbach's *α*s > .77), and high robustness from Monte Carlo simulations. Additionally, the researchers reported that the short forms constructed using penalised regression exhibited superior psychometric properties (i.e., stronger correlations with the full form, higher internal consistencies, lower standard errors) compared to those developed using Item Response Theory (IRT), a traditional approach for item reduction. The syntax of the current data analyses was adapted from Langener et al.'s (2022) study, their original syntax can be accessed at https://osf.io/3d94k.

Kramer and Huizenga (2023) further investigated one of the short forms developed by Langener et al. (2022) and found it to have favourable psychometric properties, supporting its validity as a viable alternative to the full SPM. Additionally, one of the short forms developed by Langener et al. (2022) also showed good internal reliability in a longitudinal dataset with Grade 7 students (*n* = 844; Yip & Wong, 2024), with a Cronbach's *α* of .81. These findings collectively support the effectiveness of penalised regression in developing short forms for the CPM, as evidenced by its successful implementation in creating a short form for the closely related SPM.

### Development

The current data was divided randomly into a development (70%; *n* = 235) and a validation (30%; *n* = 101) set. The development set was used to create the best CPM short form, while the validation set provided an independent evaluation of the measure's performance. This ensured that the short form developed was not excessively tailored to the development data and could provide evidence for its robustness and generalisability.

To obtain the optimised mixing (*α*) and penalty (*λ*) parameters that yield the best CPM short forms, the development set was further divided equally into a train (35%) and a test (35%) set for cross‐validation. A search space of different combinations of *α* (ranging from .4 to 1) and *λ* (ranging from 1 to 3) was applied to train the penalised regression model on the train set, using the R package Glmnet (Friedman et al., 2010). This resulted in short forms of different lengths and items, which were then evaluated for their correlation with the total score on the test set. Among short forms of the same length, the one with the highest correlation with the total score on the test set was selected. The final length of the short form was determined by an a priori rule, by considering the next longer short form until the percentage gained in correlation with the total score on the test set was below 1%.

### Validation

The final short form obtained from the item selection algorithm was subjected to multiple validation measures to assess its performance and generalisability through establishing its association with the full form, internal reliability, item stability, content validity, and concurrent validity.

To ensure the comparability of the final short form with the full 36‐item CPM, the correlation between the final short form score and the total score was examined in the validation set. This analysis aimed to assess the extent to which the scores obtained from the short form align with the overall performance on the full CPM. Additionally, the internal consistency of the final short form was examined to evaluate the reliability of the items in measuring the intended construct. This aimed to ensure a high level of agreement between the items within the final short form, such that they are measuring the same construct consistently.

To examine the stability of the items selected for the final short form, a Monte Carlo simulation was conducted using the development set. The development set was divided into different random train/test halves, and the short form selection algorithm was reapplied for each train/test randomisation to generate the best short form of the same length as the final short form. This process was repeated 100 times to obtain robust estimates of how frequently the items chosen for the final short form appeared in the generated short forms across the different train/test randomisations. The purpose of this analysis is to provide evidence for the consistency and stability of the item selection procedure and to determine whether the items emerged consistently as optimal choices for inclusion in the short forms. This may further support the importance and relevance of the items selected for the final short form.

To investigate the performance of the final short form, a comparative analysis was conducted between the final short form and 100 sets of short forms of the same length with randomly selected items. The randomly generated short forms were then correlated with the total score on the validation set. By comparing these correlation coefficients with that of the final short form, the effectiveness of the final short form in representing the total CPM score could be evaluated. Additionally, the internal consistency levels of the randomly generated short forms were compared to that of the final short form, allowing for an assessment of whether the final short form exhibited superior reliability in measuring the intended construct.

To evaluate the content validity of the final short form, the content representations of the short form items were reviewed based on the established classifications of the cognitive demands outlined in the CPM manual (Raven et al., 1998). By comparing the item distribution between the full CPM and the final short form in terms of cognitive demands, this analysis aimed to examine the alignment between the items included in the final short form and those in the full form. By evaluating the representativeness of the final short form items in relation to the broader range of cognitive demands measured by the full CPM, this analysis provided valuable insights into the adequacy and appropriateness of the items selected for the final short form.

To assess the concurrent validity of the final short form, correlations between the final short form and tasks measuring related cognitive abilities (i.e., working memory, inhibitory control, verbal knowledge, RAN) were examined in the validation set. These correlations were then compared to those reported in previous studies that investigated the relationships between the full CPM and similar constructs. By assessing the consistency of the current results with findings from existing literature, this analysis aimed to strengthen the evidence regarding the accuracy of the final short form in capturing the intended construct measured by the full CPM.

## RESULTS

**FIGURE 1:**
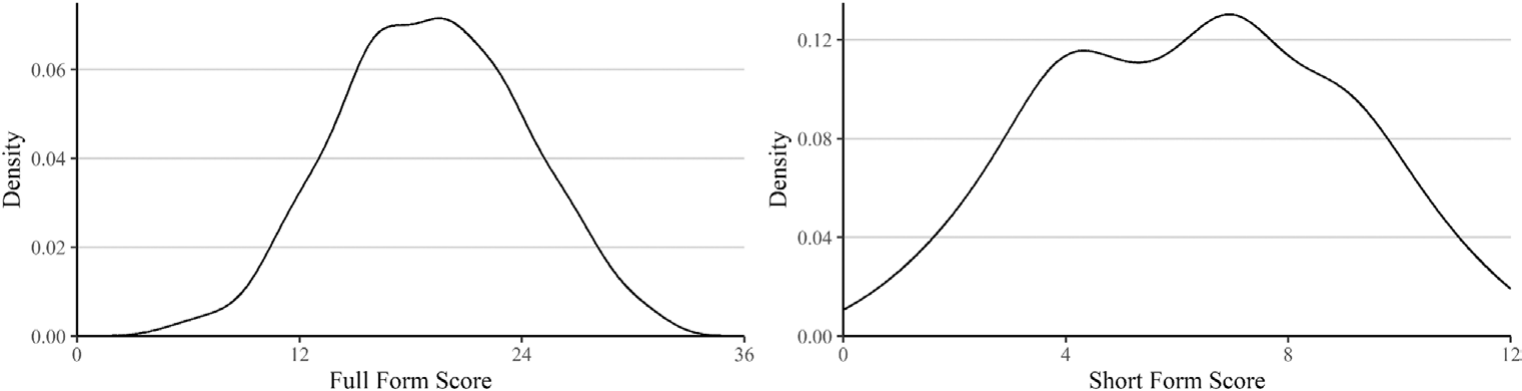
Density of CPM full form and final short form scores.

The final short form consists of 12 items selected from the original 36‐item CPM, namely A10, Ab5, Ab6, Ab7, Ab8, Ab9, B2, B4, B5, B7, B8, and B11. Table 1 presents the proportion of correct answers for all 36 CPM items in the whole sample. The proportion of correct answers of the items in the final short form ranged from .14 to .80. Figure 1 illustrates the density plots of CPM full form and short form scores. The final short form exhibited shorter tails compared to the full form and had a distribution similar to the middle area of the full form. These characteristics are typical for short forms of measures (Langener et al., 2022), as they provide a concise representation of the full form that captures its central tendency and variability within a narrower range. These suggest good comparability between the two forms.

**TABLE 1:** Proportion of correct answers for the CPM items.

Items	*p*	Items	*p*	Items	*p*
A1	.98	Ab1	.90	B1	.98
A2	.99	Ab2	.87	**B2**	.**70**
A3	.99	Ab3	.84	B3	.74
A4	.98	Ab4	.69	**B4**	.**80**
A5	.89	**Ab5**	.**77**	**B5**	.**59**
A6	.87	**Ab6**	.**68**	B6	.50
A7	.55	**Ab7**	.**63**	**B7**	.**39**
A8	.62	**Ab8**	.**36**	**B8**	.**14**
A9	.58	**Ab9**	.**47**	B9	.15
**A10**	.**61**	Ab10	.38	B10	.22
A11	.13	Ab11	.51	**B11**	.**15**
A12	.10	Ab12	.27	B12	.09
Set A	.69	Set Ab	.61	Set B	.45

*Note*: Bold indicates items included in the final short form.

During the development process, an a priori rule was applied to determine the length of the final short form. Longer short forms were considered until the percentage gained in correlation with the total score on the test set fell below 1%. Table 2 presents an overview of the various short forms considered during the development process, including the corresponding mixing (*α*) and penalty (*λ*) parameters, the short form items, their correlation with the total score on the test set, and the percentage gained in correlations. The mixing (*α*) and penalty (*λ*) parameter values of the final short form were .85 and 1.45, respectively.

**TABLE 2:** Overview of the various short forms considered during the development process.

Length	Items	Mixing *α*	Penalty *λ*	*r*	% change in *r*
2	Ab6 B5	.7	3	.63	
3	Ab6 Ab7 B5	.9	2.15	.69	8.34%
4	Ab5 Ab6 Ab7 B5	.65	2.95	.75	8.78%
5	Ab5 Ab6 Ab7 Ab9 B5	.75	2.35	.79	5.78%
6	Ab5 Ab6 Ab7 Ab9 B4 B5	.55	2.9	.81	2.46%
7	Ab5 Ab6 Ab7 Ab8 Ab9 B4 B5	.7	2.05	.83	3.25%
8	Ab5 Ab6 Ab7 Ab8 Ab9 B4 B5 B7	.65	2.25	.83	−0.17%
10	Ab5 Ab6 Ab7 Ab8 Ab9 B2 B4 B5 B7 B8	.5	2.65	.88	5.33%
11	A10 Ab5 Ab6 Ab7 Ab8 Ab9 B2 B4 B5 B7 B8	.45	2.95	.88	0.49%
**12**	**A10 Ab5 Ab6 Ab7 Ab8 Ab9 B2 B4 B5 B7 B8 B11**	.**85**	**1.45**	.**90**	2.03%
13	A10 Ab5 Ab6 Ab7 Ab8 Ab9 Ab11 B2 B4 B5 B7 B8 B11	.4	3	.91	**0.95%**
14	A8 A10 Ab5 Ab6 Ab7 Ab8 Ab9 Ab11 B2 B4 B5 B7 B8 B11	.4	2.75	.92	0.77%
15	A8 A10 Ab5 Ab6 Ab7 Ab8 Ab9 Ab11 Ab12 B2 B4 B5 B7 B8 B11	.4	2.3	.92	0.97%
16	A8 A10 Ab1 Ab5 Ab6 Ab7 Ab8 Ab9 Ab11 Ab12 B2 B4 B5 B7 B8 B11	.55	1.5	.93	0.38%

*Note*: *r* = correlation in the test set. Bold indicates final short form.

The a priori rule was satisfied with the 10‐item short form, where an addition of an item to 11 items resulted in a 0.49% increase in correlation. However, when considering the addition of items from 11 to 12, there was a 2.03% increase in the correlation, where the correlation gain remained below 1% for subsequent additions. It is also worth noting that adding an item to the 7‐item short form resulted in a 0.17% decrease in the correlation, which also falls within the range of gains below 1%. Given the greater stability in correlation gains observed beyond 12 items, the decision was made to select the 12‐item form over both the 7‐ and 10‐item forms.

### Validation

The correlation between the short form score and the total score was very strong and positive (*r* = .94, 95% BCa CI [.92, .96], *p* < .001) in the validation set. Additionally, the short form demonstrated good internal consistency (Cronbach's *α* = .80; 95% BCa CI [.75, .84]) in the validation set, compared to Cronbach's *α* = .86 (95% BCa CI [.83, .89]) for all CPM items in the validation set.

**FIGURE 2:**
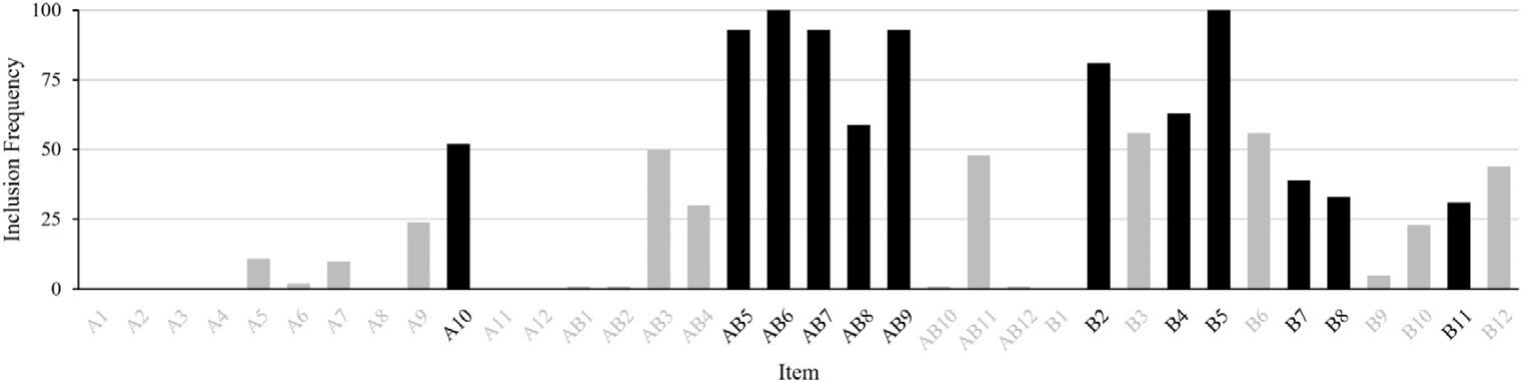
Inclusion frequency of CPM items in a 100‐run Monte Carlo simulation. Items included in the final short form are coloured in black.

The stability of the items chosen in the final short form was examined with a Monte Carlo simulation. Figure 2 illustrates the frequency with which items were included in short forms over 100 simulated models. Among the items in the final short form, Ab6 and B5 were consistently selected in 100% of the simulated models, while B11 had the lowest selection rate at 31%. Notably, nine out of the 12 items in the final short form were selected as short form items in more than 50% of the simulated runs. These findings indicated a high level of consistency and stability in the selection of items for the final short form, as demonstrated by the inclusion rates of the current short form items in the simulated models.

**FIGURE 3:**
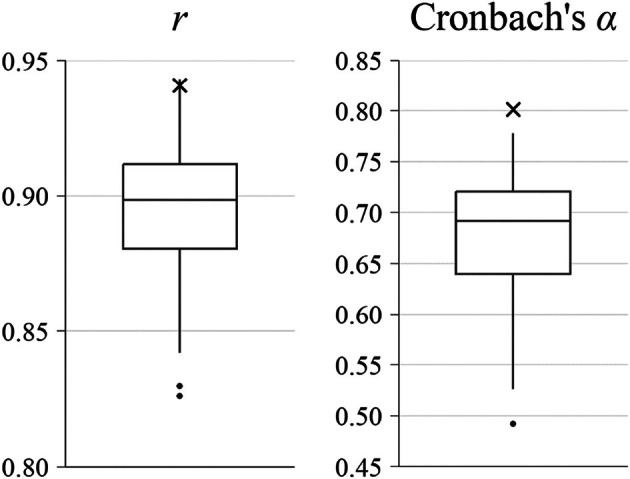
Distribution of correlations and Cronbach's *α* among randomly generated 12‐item short forms. Corresponding values of the final short form in the validation set are indicated by crosses.

The performance of the final short form was further investigated by comparing its correlation with the total score to 100 sets of 12‐item short forms with randomly selected items. Figure 3 illustrates the distribution of correlations and Cronbach's *α* between the randomly generated short forms and the total score in the validation set. The correlations ranged from .83 to .94, with a mean correlation of .89. Importantly, the final short form, which correlated with the full form at *r* = .94, outperformed 99% of the randomly generated short forms in terms of its correlation with the total score. The final short form also exhibited higher levels of internal consistency (Cronbach's *α* = .80) compared to all randomly generated short forms, with a mean Cronbach's *α* of .68 (range = .49 to .78). These results underscore the effectiveness of penalised regression in developing the current CPM short form, in contrast to randomly selected item subsets.

The content validity of the final short form was evaluated by examining the content representations for the final short form. According to the CPM manual (Raven et al., 1998), the three 12‐item sets can be further categorised into various domains representing different cognitive demands associated with the items. Table 3 summarises the distribution of the full and final short form items among the domains of cognitive demands tested based on the classification from the CPM manual (Raven et al., 1998). There were three domains without item representation from the final short form, including completion of simple continuous patterns (Set A), completion of continuous patterns showing progressive changes in two directions (Set A), and completion of discrete identical patterns (Set Ab). Excluding these domains not covered by the final short form, content representation of the final short form items was in similar proportions with that of the full CPM. Considering that the domains without item representation from the final short form contain mostly items with either a very high or very low proportion of correct responses, and that the rest of the domains are adequately represented in the final short form, the content validity of the final short form was supported by its content representation across different CPM domains of cognitive demand.

**TABLE 3:** Distribution of cognitive demands tested by CPM full form and short form items.

Domain of cognitive demand^a^	Full form	Short form
Set A: Apprehension of identity and change in *continuous patterns*	12	1
Completion of simple patterns (A1–A8)	8	0
Completion of patterns showing progressive changes in one direction (A9–A10)	2	1
Completion of patterns showing progressive changes in two directions (A11–A12)	2	0
Set Ab: Apprehension of *discrete figures* as spatially related wholes	12	5
Completion of discrete identical patterns (Ab1–Ab3)	3	0
Completion of discrete patterns as a related whole (Ab4–Ab12)	9	5
Set B: Apprehension of analogous changes in *spatially and logically related figures*	12	6
Completion of discrete identical patterns (B1–B2)	2	1
Completion of discrete patterns as a related whole (B3–B5)	3	2
Concrete or coherent reasoning by spatial analogy (B6–B9)	4	2
Discrete or abstract reasoning by logical analogy (B10–B12)	3	1

^a^ Raven et al. (1998).

The concurrent validity of the final short form was assessed using Pearson correlation coefficients between its total score and related cognitive abilities. Table 4 summarises the correlation coefficients and the corresponding correlation sizes reported by respective supporting literatures for the concurrent validity indicators. The results showed significant positive correlations between the total score of the final short form and measures of verbal working memory span, *r*(99) = .44, *p* < .001, inhibitory control, *r*(90) = .41, *p* < .001, and receptive vocabulary knowledge, *r*(73) = .50, *p* < .001. Additionally, the total score showed a non‐significant negative correlation with RAN object inverse efficiency scores, *r*(70) = −.14, *p* > .05. These correlation values were consistent with findings from previous studies (Fung et al., 2019; Lervåg & Hulme, 2010; Memisevic et al., 2023; Ng et al., 2021; Traverso et al., 2022), providing evidence for the concurrent validity of the final short form. The comparable correlation coefficients suggest that the final short form maintains similar relationships with these cognitive measures as the original full form did in previous research.

**TABLE 4:** Correlations between the final short form and related cognitive abilities.

Construct	*r* with CPM short form [95% BCa CI]	*r* in supporting literature
Verbal working memory	.44*** [.29, .57]	.42*** (Memisevic et al., 2023) .41*** (Traverso et al., 2022)
Inhibitory control	.41*** [.22, .54]	.43*** (Traverso et al., 2022) .33*** (Ng et al., 2021)
Receptive vocabulary knowledge	.50*** [.31, .64]	.43*** (Traverso et al., 2022) .42*** (Fung et al., 2019)
RAN object (IES)	−.14 [−.30, .12]	−.18* (Memisevic et al., 2023) −.19** (Lervåg & Hulme, 2010)

Abbreviations: IES, inverse efficiency score; RAN, rapid autonomised naming.

*** *p* < .001.

** *p* < .01.

* *p* < .05.

## DISCUSSION

The current study developed a 12‐item short form for the Raven's Coloured Progressive Matrices (CPM). The short form was developed using penalised regression, which has demonstrated to be an appropriate method for scale reduction, as supported by excellent correlations with the full form and item quality in previous research on short forms for the Raven's Standard Progressive Matrices (SPM; Kramer & Huizenga, 2023; Langener et al., 2022). The CPM short form exhibited a strong association with the full form (*r* = .94) and good internal consistency (Cronbach's *α* = .80), which is consistent with previous population‐based studies (Agnoli et al., 2012; Costenbader & Ngari, 2001; Cotton et al., 2005; Kazem et al., 2007). Overall, the current short form displayed strong psychometric properties and presented itself as a reliable and valid alternative to the full form.

### The final short form

The current results not only confirmed a strong relationship between the final short form and the full form, as indicated by the high correlation in between, but also supported the stability of item inclusion in the short form. Specifically, a Monte Carlo simulation revealed that most short form items were consistently selected across different random splits of the development dataset, emphasising the reliability of the item selection process. Additionally, the effectiveness of the short form was demonstrated by its superior performance in its correlation with the full form and internal consistency, in comparison to other short forms of the same length with randomly selected items. The content validity of the short form was further underscored by its comprehensive coverage across most domains of cognitive demands measured by the CPM, along with the proportional representation of these domains with reference to the full form. Lastly, concurrent validity of the final short form was supported by similar magnitudes of correlations between it and various cognitive abilities, in comparison to those reported in the literature between the full form and the abilities.

Despite the consistent selection of short form items demonstrated by the Monte Carlo simulation, it is worth noting that the simulated results revealed other CPM items with similar frequencies of inclusion, such as B3 and B6, which appeared in over half of the runs. This suggests there may be alternative short forms with comparable performance patterns that associate with the total score equally well (Langener et al., 2022). This finding echoes with previous research that has identified multiple valid short form options within the Raven's Progressive Matrices test series (Arthur & Day, 1994; Bilker et al., 2012; Bors & Stokes, 1998). Nonetheless, the current short form represents a specific selection of items that demonstrated consistent performance and excellent correlation with the CPM full form.

Previous research conducted with older adults suggested that Set Ab of the CPM had minimal contribution to differentiating cognitive performance when compared to Sets A and B (Levinson, 1959; Smits et al., 1997). However, contrary to this perspective, the current short form included five out of 12 items from Set Ab. These items were retained in the short form by penalised regression models based on their importance and contribution to the total score (Zou & Hastie, 2005). Specifically, the retention of these items signifies their value and relevance in explaining the variation in the outcome variable (i.e., the total score), while other items are considered less influential or redundant in predicting the outcome variable (Zou & Hastie, 2005). Although Set Ab items may have limited relevance in certain populations, such as the older adults (Levinson, 1959; Smits et al., 1997), their significance appears to hold true for typically developing preschoolers in the current study. Further research could explore the cognitive processes and developmental factors associated with the performance of Set Ab to gain insights into its utility for measuring cognitive abilities across different populations.

Concurrent validity of the current short form was corroborated by similar levels of correlation between the current short form and various cognitive abilities, compared to those reported between these cognitive abilities and the full form in the literature. This suggests that the short form is effective in capturing the construct measured by the full form, further supporting its use as a viable alternative to the lengthier full version. Since Raven's Progressive Matrices are often used as a proxy measure of intelligence (Alhamdan et al., 2023; Carvalho et al., 2022; Gofer‐Levi et al., 2014; Memisevic et al., 2023; Ng et al., 2021; Ouyang et al., 2023), and these tests are frequently employed as screening tools across research and practice (Kashani‐Vahid et al., 2017; Ma & Jia, 2024; Yip et al., 2020), it is also important to directly compare the current short form and gold standard intelligence measures (e.g., Wechsler Intelligence Scales, Stanford–Binet Intelligence Scales).

In the current study, among the concurrent validity indicators, the backwards digit span (BDS) task had a similar design and administration to the digit span subtest of the Wechsler Intelligence Scale for Children (WISC). Additionally, the Hong Kong Cantonese Receptive Vocabulary Test (CRVT) is a close language variant of the Peabody Picture Vocabulary Test (PPVT), which has been shown to strongly correlate with the Verbal IQ Score in WISC (*r*s > .76; Campbell, 1998; Carvajal et al., 1993). While the BDS and CRVT may be considered proxies of corresponding subtests and indices of WISC, certain WISC components (e.g., processing speed) were not included in the current study. Future researchers should explore the associations between the current short form and other WISC subtests to extend the construct validity of the current short form.

Similar efforts were made by Arthur and Day (1994), who included measures strongly correlated with the Wechsler Adult Intelligence Scale (WAIS) in their evaluation of an APM short form. However, such investigations were not common in studies developing short forms for Raven's Progressive Matrices (Bilker et al., 2012; Bors & Stokes, 1998; Langener et al., 2022), although one study (Hall, 1957) did report moderate‐to‐strong correlations between an SPM short form and WAIS subtests. Future studies developing short forms for the Raven's Progressive Matrices should incorporate gold standard intelligence measures to assess the validity of the short forms.

### Significance

The current study represented one of the first initiatives to create a short version of the CPM specifically tailored for the preschool population that utilises the whole measure. In addition to its satisfactory psychometric properties, the final short form demonstrated comparative effectiveness when compared to other published versions of abbreviated CPM forms. In the current validation sample, the 18‐item short form developed by Lúcio et al. (2019) for younger children showed lower internal consistency (Cronbach's *α* = .75; 95% BCa CI [.66, .82]) and weaker correlations with the full CPM (*r* = .88). Similarly, the 24‐item short form developed by Smits et al. (1997) for older adults exhibited slightly lower internal consistency (Cronbach's *α* = .78; 95% BCa CI [.72, .83]), but a slightly stronger correlation (*r* = .95) with the full form. Nevertheless, despite containing only half the number of items compared to Smits et al.'s (1997) version, the current short form maintained a comparable level of correlation with the full form, indicating its high efficiency. This highlights the strength of the final short form in representing the full CPM for preschoolers, while other abbreviated versions of CPM may be more suitable for different populations.

The current short form of the CPM has significantly reduced the number of items, shortening it to one‐third of its original length. This reduction has important practical implications for researchers planning to use the measure, as it can substantially reduce the time and resources required to administer the CPM. Considering that the CPM is frequently used in research studies to assess nonverbal intelligence in young children as a background variable (Alhamdan et al., 2023; Carvalho et al., 2022; Gofer‐Levi et al., 2014; Memisevic et al., 2023; Ng et al., 2021; Ouyang et al., 2023), introducing a short form may afford researchers to adopt a more efficient and practical research design. By utilising a shorter version of the CPM, researchers may allocate additional time and resources to explore and measure other variables of interest, thereby expanding the scope and depth of their investigation. Moreover, the short form represents a promising alternative to the full CPM, particularly in large‐scale studies, as it enables researchers to streamline data collection and reduce participant burden without significantly compromising the quality of data on participants' nonverbal cognitive abilities. To summarise, the introduction of the CPM short form may not only increase the efficiency of data collection procedures but also provide new opportunities for researchers to conduct more comprehensive and nuanced studies.

### Limitations

The current study was not without limitations. First, the current short form was developed with a late preschooler sample, which may limit the generalisability of the current short form to older children, as well as other populations that utilise the CPM, such as the elderly or individuals with mental impairments. Nevertheless, researchers may utilise other available short forms of the Raven's Progressive Matrices when assessing older children (e.g., SPM short forms; Langener et al., 2022) and the elderly (e.g., CPM short forms; Smits et al., 1997). Future studies may also validate the current short form with different age groups or develop specific CPM short forms for different populations.

Relatedly, the current sample was recruited from local preschools in Hong Kong, where the cultural homogeneity may limit the generalisability of our findings to children from other cultural backgrounds. Given prior research suggesting cultural differences in the performance of Raven's tests (Gonthier, 2022; cf. Raven et al., 1998), future researchers should validate the current short form in other populations to confirm the universal applicability of our findings.

Second, the full 36‐item version of the CPM was administered during the current data collection, which may result in different response patterns for the short form items when compared to their independent administration. Specifically, Raven's Progressive Matrices was designed with the assumption that the participants learn from experience throughout the test, as the difficulty level progresses from simpler to more complex items within each set (Raven et al., 1998). Consequently, a reduced number of test items in the short form may result in fewer insights gained from the earlier items, potentially lowering overall performance (Langener et al., 2022). To mitigate this issue to some extent, it is recommended to administer suitable practice items (e.g., A1 and A2) before proceeding to the short form items, as well as to maintain the original order presentation. This ensures participants are adequately familiarised with the test format and provided with sufficient learning opportunities. Nevertheless, it remains crucial for future research to thoroughly evaluate the psychometric properties of the short form when administered independently, comparing its performance to both the full version and the current embedded short form.

Third, the individual scores of the current short form have limited interpretability (e.g., to be converted into standardised IQ scores), since the current study was not able to calculate reliable norm references with a limited sample size. It is also not advisable to adapt long‐form norms to interpret individual scores of short forms (Arthur & Day, 1994). To enable comparison and interpretation of individual scores, future researchers may conduct normative studies specifically for the current short form to derive representative norms.

## CONCLUSIONS

In conclusion, the current study developed a 12‐item short form for the Raven's Coloured Progressive Matrices (CPM) using a machine learning approach. This short form demonstrated very strong correlation with the total score of the CPM, along with good internal consistency, item stability, content validity, and concurrent validity. The reduced number of items in the short form allows researchers to assess children's nonverbal cognitive abilities more efficiently with significantly less administration time. This development creates new possibilities for researchers to expand the scope and depth of their research endeavours, contributing to the progress of both research and practice across various fields of studies.

## AUTHOR CONTRIBUTIONS

**Charles Chiu Hung Yip:** Conceptualization; data curation; formal analysis; methodology; project administration; visualization; writing – review and editing; writing – original draft. **Terry Tin‐Yau Wong:** Conceptualization; methodology; supervision; writing – review and editing. **Brandon Hoi Dick Wong:** Conceptualization; data curation; methodology; writing – review and editing; writing – original draft. **Lucy Shih‐Ju Hsu:** Data curation; investigation; funding acquisition; supervision; writing – review and editing.

## FUNDING INFORMATION

This work was supported by a fund awarded to LH from the University Research Committee (URC) of the University of Hong Kong.

## CONFLICT OF INTEREST STATEMENT

The authors declared no potential conflicts of interest with respect to the research, authorship, and/or publication of this article.

## Data Availability

Study data and analysis scripts are available upon reasonable request from the corresponding author.
